# Study of AC Conductivity and Relaxation Times Depending on Moisture Content in Nanocomposites of Insulation Pressboard–Innovative Bio-Oil–Water Nanodroplets

**DOI:** 10.3390/ma17235767

**Published:** 2024-11-25

**Authors:** Pawel Zukowski, Konrad Kierczynski, Pawel Okal, Marek Zenker, Rafal Pajak, Marek Szrot, Pawel Molenda, Tomasz N. Koltunowicz

**Affiliations:** 1 Lublin University of Technology, 38d, Nadbystrzycka Street, 20-618 Lublin, Poland; zukowski50pawel@gmail.com; 2 Department of Electrical Devices and High Voltage Technology, Lublin University of Technology, 38a, Nadbystrzycka Street, 20-618 Lublin, Poland; k.kierczynski@pollub.pl (K.K.); t.koltunowicz@pollub.pl (T.N.K.); 3 Department of High Voltage and Power Engineering, West Pomeranian University of Technology, 37, Sikorskiego Street, 70-313 Szczecin, Poland; marek.zenker@zut.edu.pl; 4 Nynas AB Raffinaderivagen, 21, Nynashamn Stoc, 149 82 Nynasham, Sweden; rafal.pajak@nynas.com; 5 Energo-Complex, Sp. z o.o., 9, Lotników Street, 41-949 Piekary Śląskie, Poland; marek.szrot@energo-complex.pl (M.S.); pawel.molenda@energo-complex.pl (P.M.)

**Keywords:** power transformer, electrical properties, bio-oil, moisture, cellulose, insulating oil

## Abstract

The aim of this study was to determine the frequency–temperature dependence of the AC conductivity and relaxation times in humid electrical pressboard used in the insulation of power transformers, impregnated with the innovative NYTRO^®^ BIO 300X bio-oil produced from plant raw materials. Tests were carried out for a composite of cellulose–bio-oil–water nanodroplets with a moisture content of 0.6% by weight to 5% by weight in the frequency range from 10^−4^ Hz to 5·10^3^ Hz. The measurement temperatures ranged from 20 °C to 70 °C. The current conductivity in percolation channels in cellulose–bio insulating oil–water nanodroplets nanocomposites was analyzed. In such nanocomposites, DC conduction takes place via electron tunneling between the potential wells formed by the water nanodroplets. It was found that the value of the percolation channel resistance is lowest in the case of a regular arrangement of the nanodroplets. As disorder increases, characterized by an increase in the standard deviation value, the percolation channel resistance increases. It was found that the experimental values of the activation energy of the conductivity and the relaxation time of the composite of cellulose–bio-oil–water nanodroplets are the same within the limits of uncertainty and do not depend on the moisture content. The value of the generalized activation energy is Δ*E* ≈ (1.026 ± 0.0160) eV and is constant over the frequency and temperature ranges investigated. This study shows that in the lowest frequency region, the conductivity value does not depend on frequency. As the frequency increases further, the relaxation time decreases; so, the effect of moisture on the conductivity value decreases. The dependence of the DC conductivity on the moisture content was determined. For low moisture contents, the DC conductivity is practically constant. With a further increase in water content, there is a sharp increase in DC conductivity. Such curves are characteristic of the dependence of the DC conductivity of composites and nanocomposites on the content of the conducting phase. A percolation threshold value of *x*_c_ ≈ (1.4 ± 0.3)% by weight was determined from the intersection of flat and steeply sloping sections. The frequency dependence of the values of the relative relaxation times was determined for composites with moisture contents from 0.6% by weight to 5% by weight for a measurement temperature of 60 °C. The highest relative values of the relaxation time *τ*_ref_ occur for direct current and for the lowest frequencies close to 10^−4^ Hz. As the frequency increases further, the relaxation time decreases. The derivatives *d*(*logτ*_ref_)/d(log*f*) were calculated, from the analysis of which it was determined that there are three stages of relaxation time decrease in the nanocomposites studied. The first occurs in the frequency region from 10^−4^ Hz to about 3·10^−1^ Hz, and the second from about 3·10^−1^ Hz to about 1.5·10^1^ Hz. The beginning of the third stage is at a frequency of about 1.5·10^1^ Hz. The end of this stage is above the upper range of the Frequency Domain Spectroscopy (FDS) meter, which is 5·10^3^ Hz. It has been established that the nanodroplets are in the cellulose and not in the bio-oil. The occurrence of three stages on the frequency dependence of the relaxation time can be explained when the fibrous structure of the cellulose is taken into account. Nanodroplets, found in micelles, microfibrils and in the fibers of which cellulose is composed, can have varying distances between nanodroplets, determined by the dimensions of these cellulose components.

## 1. Introduction

The primary components of power transformers are windings, a ferromagnetic core, and insulating materials. This configuration of transformer elements has remained unchanged since the invention of transformers at the end of the 19th century. However, significant changes have occurred over time in the materials used to construct the primary components, particularly concerning the core material. On the other hand, the insulating materials remain practically unchanged. These include cellulose and mineral insulating oil [1,2]. Cellulose materials perform two main functions in power transformers. First, they insulate components with a significant voltage difference. Second, they act as structural elements, ensuring the mechanical strength of the winding and core assembly.

The second insulating factor is oil. The primary functions of the oil are to improve the insulating properties of the system, including increasing its resistance to high voltages. This occurs in oil channels, which are placed between the solid insulation and through the impregnation of paper and pressboard. Impregnating cellulose with oil enhances its insulating parameters [3] and slows down its aging processes [4,5,6], thereby extending the lifespan of the insulation. Additionally, the oil in power transformers functions as a coolant, absorbing heat from the active parts and dissipating it externally. The circulation speed of the oil is determined by its kinematic viscosity.

Currently, in the manufacturing process of power transformers, all components are produced. They are then assembled in a tank. After the tank is hermetically sealed, the cellulose insulation undergoes vacuum drying at elevated temperatures. This is necessary because cellulose absorbs moisture from the air, with its content potentially reaching up to 8% by weight. Such cellulose is unsuitable for insulation due to its unsatisfactory resistivity. After vacuum drying, the moisture content is reduced to 0.8% by weight or lower [7,8,9]. Next, the transformer is filled with insulating oil heated to a temperature above 60 °C, also in a vacuum. The elevated temperature and vacuum facilitate the rapid impregnation of cellulose, as the kinematic viscosity of the oil decreases several times with an increase in temperature.

As transformers age, the moisture content in the cellulose gradually increases. Moisture precipitates from the cellulose during aging processes. However, the moisture caused by this factor is relatively low and usually does not exceed a few tenths of a percent [10,11]. The second source of increased moisture content is its slow penetration from the outside.

Water accumulates in the cellulose, which is related to its solubility in cellulose being several orders of magnitude higher than in oil [2]. A moisture content of approximately 5% by weight is considered the critical threshold for paper–oil insulation [2,12,13]. Reaching or exceeding the critical value can lead to transformer failure. This indicates that to prevent failure, it is necessary to periodically monitor the moisture content of the cellulose. Electrical methods, both direct current [14,15] and alternating current [16,17,18], are used for diagnosing the condition of the insulation, including water content [19,20,21,22]. In direct current measurements, the Polarization Depolarization Current (PDC) method determines the direct current resistance and relaxation times [14,15]. The Return Voltage Measurement (RVM) method [16,17,18] assesses relaxation times based on moisture levels. Currently, the most commonly used method for diagnosing insulation condition is Frequency Domain Spectroscopy (FDS) [19,20,21,22].

Diagnosis using the aforementioned methods—PDC, RVM, and FDS—requires the prior determination of so-called reference characteristics, which describe the relationships between the measured parameters and moisture content and temperature (see, for example, [23]). For this purpose, it is essential to conduct appropriate laboratory tests. To accurately determine the moisture content, models that faithfully describe the physical phenomena related to the flow of direct or alternating current in moisture-containing cellulose impregnated with insulating oil should also be applied.

In a series of articles (see, for example [24,25]), it has been established that moisture in the solid insulation component self-assembles into nanodroplets containing up to several hundred water molecules. Consequently, moisture-laden cellulose impregnated with oil should be treated as a nanocomposite consisting of pressboard, oil, and nanodroplets of water. This means that the analysis of current conduction in such materials should be based on the quantum mechanical phenomenon of electron tunneling between the water nanodroplets.

In recent decades, new types of insulating oils have been developed, produced from plant-based raw materials. The primary aim of their production and use in power transformers is to reduce the consumption of non-renewable fossil resources. This decreases the so-called carbon footprint in the production of power transformers. Furthermore, bio-oils are characterized by very good biodegradability [26,27], which eliminates potential environmental contamination. Among these oils is NYTRO^®^ BIO 300X (Nynas AB Raffinaderivagen, Nynashamn, Sweden). NYTRO^®^ BIO 300X has slightly different electrical parameters compared to petroleum-based insulation oils [5,27,28]. It follows that to diagnose the condition of cellulose insulation, it is necessary to determine the reference electrical parameters, including the relationships of conductivity and relaxation times with temperature and moisture content in pressboard impregnated with NYTRO^®^ BIO 300X.

The purpose of this work is to determine the frequency–temperature relationships of alternating current conductivity and relaxation times in moisture-laden electrical pressboard used in the insulation of power transformers, impregnated with the innovative bio-oil NYTRO^®^ BIO 300X, produced from plant-based raw materials.

## 2. Resistance of the Percolation Channel with Nanodroplets—Theoretical Foundations

In studies [29,30], it has been shown that moisture in electrical pressboard impregnated with insulating oil self-assembles into nanodroplets containing, on average, several hundred water molecules. For direct current conduction to occur in the nanocomposite, at least one percolation channel must be formed, connecting the contacts to which the measurement voltage is applied. In the case of composites with macroscopic grain sizes, the percolation channel is formed through the contact of adjacent grains of the conductive phase [31,32,33,34]. In nanocomposites with conductivity resulting from electron tunneling, typical representatives of which are nanocomposites with dielectric matrices containing nanoinclusions of highly conductive phases (nanoparticles of metals, semiconductors, water nanodroplets), the formation of a percolation channel does not necessarily require direct contact between the nanoscale grains of the conductive phase. Nanoparticles of the conductive phase, embedded in the insulating material, create potential wells. When the distances between potential wells are in the order of nanometers, the phenomenon of electron tunneling from one potential well to another occurs, as known from quantum mechanics [35] The conductivity resulting from tunneling between potential wells is referred to as hopping conductivity [36]. A percolation channel in a nanocomposite with hopping conductivity forms when a chain of nanoparticles is created, connecting contacts that are spaced at distances sufficient for tunneling to occur between the nearest wells.

The probability of electron tunneling per unit time *P* between three-dimensional potential wells—nearest neighbors—is described by the following formula [36]:

(1) $$P=P_{0}\exp\left(-\frac{2}{R_{B}}r\right)\exp\left(-\frac{\Delta E}{kT}\right),$$

where *P*_0_ = const—numerical coefficient; *r*—distance over which an electron tunnels; *R_B_*—the localization radius of the wave function of an electron in a potential well, commonly referred to as the Bohr radius; *ΔE*—activation energy of tunneling; *k*—Boltzmann constant; *T*—temperature.

**Figure 1:**
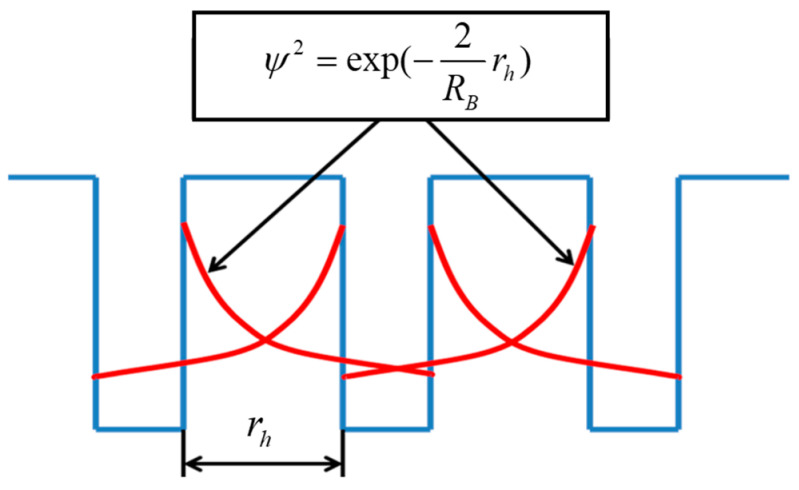
Schematic representation of the rate of decay of the square of the wave function outside a potential well with nanometer dimensions, the distance between which is *r_h_*.

The first exponent in Formula (1) describes the rate of decay of the square of the wave function of the electron outside the three-dimensional potential well. Figure 1 schematically illustrates the rate of decay of the square of the wave function outside the three-dimensional potential well and its penetration into a second well.

**Figure 2:**
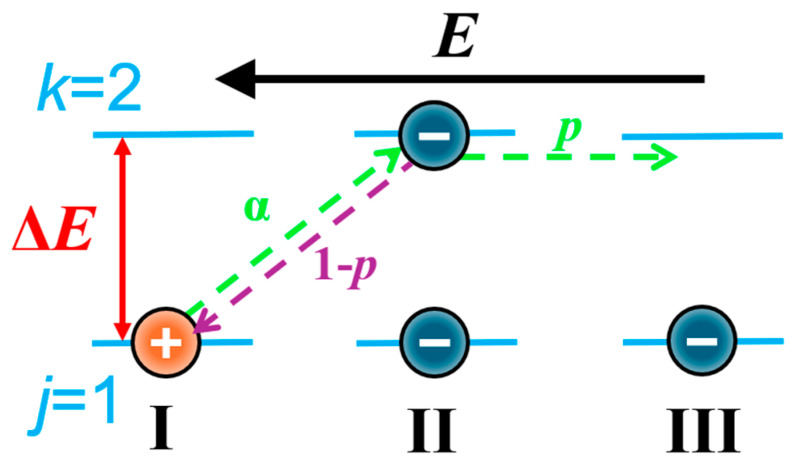
*a*—tunneling of the electron from the first well to the second well in the direction opposite to the electric field; *p*—tunneling of the electron with a probability *p* from the second well to the third well; 1 − *p*—back tunneling of the electron with a probability of 1 − *p* from the second well to the first well.

This type of tunneling is often referred to as thermally activated. The activation energy that enters into Formula (1) is related to the fact that when the potential wells are identical, the valence levels in each well are occupied by valence electrons. Consequently, the tunneling electron jumps from the first well to a higher level in the second well. This type of hopping is illustrated in Figure 2 part a. In an electric field, an asymmetry in the jumps of electrons is created in the direction aligned with the field and in the opposite direction, as defined by formula [36]:

(2) $$\exp\left(\pm \frac{erE}{kT}\right)=\sinh\left(\frac{erE}{kT}\right),$$

where *e*—electron charge; *E*—electric field strength.

This asymmetry manifests as the flow of electric current. As a result of the jump, an electric dipole is formed. From Formula (2), after an electron tunnels from the first well, it results in a vacancy of one electron in that well. The first well becomes positively charged. In the second well, an extra electron appears, resulting in a negative charge [37]. In [38], it was first demonstrated that after tunneling from the first well to the second well, the electron must remain in the latter for a certain time τ, known as the relaxation time. This is the time required for the electron to thermalize after tunneling. After time *τ*, the electron can jump with a probability *p* in the opposite direction to the electric field, moving to the third well (Figure 2 part *p*). This causes a direct current to flow. After the relaxation time, the electron has a probability of 1 − *p* (Figure 2 part 1 − *p*) to return from the second well to the first well, which leads to the decay of the dipole and results in the flow of alternating current.

From the model of hopping conductivity under DC and AC based of electron tunneling [24,38,39], it follows that for the parallel equivalent circuit, where the alternating conductivity is accurately measured, its value is as follows:

(3) $$\sigma_{r}=\sigma_{0}\left(1-\cos\varpi \tau +2p\cos\varpi \tau \right),$$

where *ω*—angular frequency; *σ*_0_—high-frequency conductivity; *p*—the probability of tunneling from the second well to the third well; *τ*—relaxation time.

From the analysis of Formula (3), it follows that for DC:

(4) $$\sigma_{r0}=2p\sigma_{0}=const,$$

The analysis of the probability of a jump from the second well to the third well (Figure 2), which is included in Formulas (3) and (4) and causes a direct current flow, showed that its value is [24]:

(5) $$p=\exp\left(-\frac{2}{R_{B}}r-\frac{\Delta E}{kT}+\frac{U_{1}}{2kT}\right),$$

where *U*_1_ which is the potential energy of the dipole (Figure 2):

(6) $$U_{1}=\frac{e}{4\pi \varepsilon \varepsilon_{0}r},$$

When conducting measurements at a constant temperature, Formula (5) can be represented as follows:

(7) $$p(T=const)=p_{A0}\exp\left(-\frac{2}{R_{B}}r\right),$$

where:

(8) $$p_{A0}(T=const)=p_{0}\exp\left(-\frac{\Delta E}{kT}+\frac{U_{1}}{2kT}\right),$$

The conduction of direct current through a material implies that charge must flow from one electrode on one side of the material to the electrode on the opposite side. For the flow of direct current through a composite, the formation of a percolation channel [32] or a nanocomposite [40,41,42,43,44,45] is essential. The percolation channel can be represented as a series connection of a large number of resistors, where adjacent potential wells play the role of resistors between which electron tunneling occurs. The resistance corresponding to this tunneling, represented by a single resistor *R*_1_, can be expressed using Formula (9):

(9) $$R_{1}(T=const)=R_{1,0}p_{A0}^{-1}\exp\left(-\frac{2}{R_{B}}r\right),$$

where *R*_1,0_—numerical coefficient.

The number of nanometer-scale potential wells forming the percolation channel, as well as the number of individual resistors *R*_1_, is very large. For a layer of nanocomposite with a length in the order of centimeters, it can be around 10^6^ or more. Assuming that the nanoparticles in the percolation channel are randomly distributed, according to the Central Limit Theorem, the distances between adjacent nanoparticles are described by a normal distribution [46]:

(10) $$F(r)=\frac{1}{\sigma_{p}\sqrt{2\pi }}\exp\left(-\frac{{\left(r-\mu \right)}^{2}}{2\sigma_{p}^{2}}\right),$$

where *F*(*r*)—probability of occurrence of distances *r* (the length of the electron’s jump during tunneling) between neighboring nanoparticles; *µ*—expected value; *σ_p_* standard deviation.

The resistance of the percolation channel for *T = const* can be expressed as follows:

(11) $$R_{p}=\sum_{i=1}^{n}R_{1i}=\frac{1}{\sigma_{p}\sqrt{2\pi }}\sum_{i=1}^{n}\exp\left(-\frac{{\left(r_{i}-\mu \right)}^{2}}{2\sigma_{p}^{2}}\right)R_{1,0}p_{0}^{-1}\exp\left(\frac{2}{R_{B}}r_{i}\right),$$

where *n*—the number of nanoparticles in the percolation channel.

Formula (11) is transformed, considering that each distance *r* between neighboring nanoparticles can be represented as follows:

(12) $$r_{i}=\mu +\delta r_{i},$$

where *μ*—the average distance between neighboring nanodroplets; *δr_i_*—the difference between the distance between nanoparticles numbered *i* and *i* + 1 and the average value *μ*.

Substituting Equation (12) into the last exponent in Equation (11) and rearranging gives us the following:

(13) $$\exp\left(\frac{2}{R_{B}}r_{i}\right)=\exp\left[\frac{2}{R_{B}}\left(\mu +\delta r_{i}\right)\right]=\exp\left(\frac{2}{R_{B}}\mu \right)\exp\left(\frac{2}{R_{B}}\delta r_{i}\right),$$

Formulas (12) and (13) substitute into Formula (11):

(14) $$R_{p}=R_{1,0}p_{0}^{-1}\exp\left(\frac{2}{R_{B}}\mu \right)\sum_{i=1}^{n}\frac{1}{\sigma_{p}\sqrt{2\pi }}\exp\left(-\frac{{\left(\delta r_{i}\right)}^{2}}{2\sigma_{p}^{2}}\right)\exp\left(\frac{2}{R_{B}}\delta r_{i}\right),$$

We will analyze the effect of the random distribution of nanoparticles in the percolation channel on its resistance. When nanoparticles form a percolation channel in the form of a regular chain, the value of *δr_i_* = 0. The value of the second exponent in equation Formula (14) equals 1, allowing us to factor it out of the sum. We are left with the sum of the probability values, which represents the cumulative distribution function of the normal distribution, and its value equals 1. In this case, the resistance of the percolation channel is as follows:

(15) $$R_{p}=nR_{1,0}p_{0}^{-1}\exp\left(\frac{2}{R_{B}}\mu \right),$$

It is probably the smallest possible resistance. Now, we will sort the values entering Formula (14) according to the values of *δr_i_* in ascending order. The normal distribution of probability density is symmetric around the mean value *μ*. This means that in each interval Δ*r*, located on both the left and right sides of the mean value *μ*, there is the same distance between nanoparticles in the percolation channel. On the left side, the values of *δr_i_* are negative, while on the right side, they are positive. Thus, the pair of these values gives the following:

(16) $$\exp\left(-\frac{2}{R_{B}}\delta r_{i}\right)+\exp\left(\frac{2}{R_{B}}\delta r_{i}\right)=2\cosh\left(\frac{2}{R_{B}}\delta r_{i}\right),$$

For very small values of δ*r*_i_, the sum of the values from Formula (16) approaches 2 because cos*h*(*x* = 0) = 1. For larger values, the sum will exceed 2. This means that the random arrangement of nanoparticles in the percolation channel results in its resistance being greater than if the nanoparticles were arranged regularly in a chain. From Equation (11), it follows that the greater the standard deviation in the normal distribution of distances between neighboring nanoparticles, the higher the resistance of such a channel. This is easy to understand considering that in a chain of series-connected resistors associated with electron tunneling between adjacent nanoparticles, the largest value is held by the resistor where the tunneling electron must overcome the greatest distance, as seen in Formula (9). In the case of the normal distribution of distances over which electrons tunnel, a numerical coefficient *A*(*σ_p_*), will appear in Formula (16), the value of which is greater than one and depends on the value of *σ_p_*:

(17) $$R_{p}=A(\sigma_{p})R_{1,0}p_{0}^{-1}\exp\left(\frac{2}{R_{B}}\mu \right)\sum_{i=1}^{n/2}\frac{1}{\sigma_{p}\sqrt{2\pi }}\exp\left(-\frac{{\left(\delta r_{i}\right)}^{2}}{2\sigma_{p}^{2}}\right)\cosh\left(\frac{2}{R_{B}}\delta r_{i}\right),$$

In Formula (17) and the preceding ones, the average distance between neighboring nanoparticles is defined by Formula [36]:

(18) $$r=\mu ≅N^{-\frac{1}{3}},$$

where *N*—the concentration of water nanodroplets.

From Formula (18), it follows that when the distance between the contacts is unitary, the number of water nanodroplets in the percolation channel is as follows:

(19) $$n=N^{\frac{1}{3}},$$

As the concentration of potential wells *N* increases, the average tunneling distance *μ*—as given in Equation (18)—and the standard deviation *σ_p_*, which is related to the expected value, decrease:

(20) $$A(\sigma_{p})∼\sigma_{p}≅c\mu ≅cN^{-\frac{1}{3}},$$

where *c*—numerical coefficient.

Substituting Formulas (18)–(20) into Formula (17), we obtain the following:

(21) $$R_{p}=R_{1,0}p_{0}^{-1}N^{\frac{1}{3}}cN^{-\frac{1}{3}}\exp\left(\frac{2}{R_{B}}N^{-\frac{1}{3}}\right)=cR_{1,0}p_{0}^{-1}\exp\left(\frac{2}{R_{B}}N^{-\frac{1}{3}}\right),$$

The formula for the direct current conductivity of the percolation channel can be expressed as follows:

(22) $$\sigma_{h}(T=const)=\frac{1}{R_{p}}=\frac{1}{cR_{1,0}p_{0}^{-1}}\exp\left(-\frac{2}{R_{B}}N^{-\frac{1}{3}}\right),$$

From Formula (22), it follows that the relationship between the direct current conductivity of the percolation channel and the concentration of nanoparticles is significantly stronger than linear. Considering the temperature dependence, the formula for conductivity in the case of hopping conductivity via electron tunneling can be reduced to the following well-known expression [36,47]:

(23) $$\sigma_{h}=\sigma_{0}\exp\left(-\frac{2}{R_{B}}N^{-\frac{1}{3}}\right)\exp\left(-\frac{\Delta E}{kT}\right),$$

## 3. Materials and Methods

For this study of the effect of moisture on conductivity and relaxation time of pressboard impregnated with bio-insulating oil, a series of 6 pressboard plates with water content of 0.6% by weight, 1% by weight, 2% by weight, 3% by weight, 4% by weight, and 5% by weight were prepared. The samples for this study were made from electrical pressboard produced by Weidmann [48]. The impregnation was carried out using the bio-oil NYTRO^®^ BIO 300X [26]. The preparation of the samples followed the standard procedure described in numerous publications (see, for example [4,49,50,51,52]). Samples with a diameter of 190 mm, cut from sheets with a thickness of 0.5 mm, were dried for approximately 3 days in a vacuum below 1 hPa at a temperature of 353 K. Then, using the Karl Fischer Titration method [53], the concentration of water remaining in the pressboard after drying was determined. Its value was around 0.6% by weight. The sample was placed on a scale, which allowed for continuous precise monitoring of its mass. Based on the sample’s mass, determined immediately after drying, and the moisture content, the mass after humidification to the moisture content of *X* % by weight was calculated. By absorbing moisture from the atmospheric air, the mass of the sample increased. After reaching the target mass, corresponding to the moisture content of *X* % by weight, the sample was placed in a vessel filled with bio-oil NYTRO^®^ BIO 300X [26]. The vessel was sealed and placed in a climate chamber. To accelerate the impregnation process, a temperature of 45 °C was applied. At this temperature, the viscosity of the bio-oil, which determines the duration of the impregnation process, is about three times lower than at a temperature of around 20 °C. The impregnation process was carried out over two weeks.

**Figure 3:**
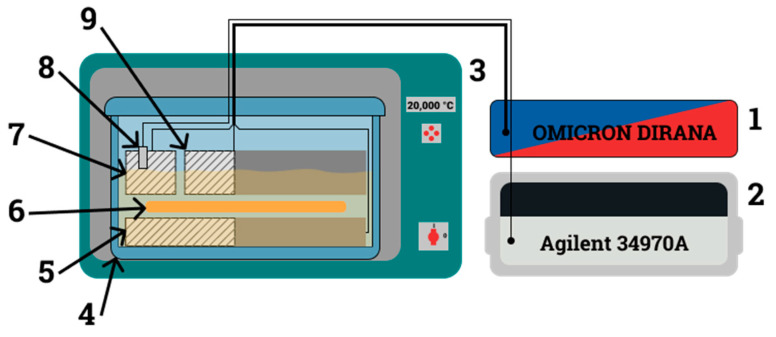
Diagram of the setup for AC measurements of the electrical properties of insulating materials: 1—FDS-PDC Dielectric Response Analyzer; 2—temperature meter; 3—climate chamber; 4—hermetic vessel; 5—voltage electrode; 6—tested sample; 7—guard electrode; 8—PT 1000 temperature sensor; 9—measurement electrode.

AC tests on the moisturized samples impregnated with bio-oil were conducted using the setup, the diagram of which is shown in Figure 3. The test setup has been described in detail in a number of publications (see, for example [54,55,56]). The basic components of the setup are the FDS-PDC Dielectric Response Analyzer [57]—1; an Agilent temperature recorder—2; a climate chamber—3; and a three-electrode capacitor—5, 7, 9. The test sample is inserted into the capacitor and placed in a glass vessel—4. The vessel was sealed and placed in a thermostat. The first temperature was set at 20 °C, and after stabilization, AC measurements were performed in the frequency range from 5·10^3^ Hz to 10^−4^ Hz. Then, the next measurement temperature was set, and the measurement cycle was repeated.

## 4. Determination of the Dependence of AC Relaxation Time on Moisture Content

The electrical properties of nanocomposites as well as other insulating materials are described by two material parameters—conductivity and dielectric permittivity. These parameters enter into Maxwell’s second equation, which describes the current flow in materials [58]. The material parameters depend, in turn, on the relaxation time. In a number of cases, the conductivity relaxation time and the permittivity relaxation time differ. This occurs when the mechanism of carrier formation and the mechanism of polarization are not related. For example, in semiconductors, carriers (electrons or holes) are formed by doping. In contrast, the polarization mechanism is independent of doping. A similar situation occurs in ionic crystals, where carriers are formed by the ionization of vacancies or interstitial atoms. The values of the conductivity and relaxation times depend on the temperature via the Debye factor [59,60]:

(24) $$\sigma =\sigma_{0}\exp\left(-\frac{\Delta E(\sigma )}{kT}\right),$$

where *σ*—conductivity; *σ*_0_—numerical factor; Δ*E*(*σ*)—activation energy of conductivity.

(25) $$\tau =\tau_{0}\exp\left(-\frac{\Delta E(\tau )}{kT}\right),$$

where *τ*—relaxation time; *τ*_0_—numerical factor; Δ*E*(*τ*)—activation energy of the relaxation time.

In dielectric materials, there may be a difference between the mechanism determining the conductivity and the mechanism determining the polarization. In this case, the values of the activation energy entering Formulas (24) and (25) will be different for conductivity and polarization.

**Figure 4:**
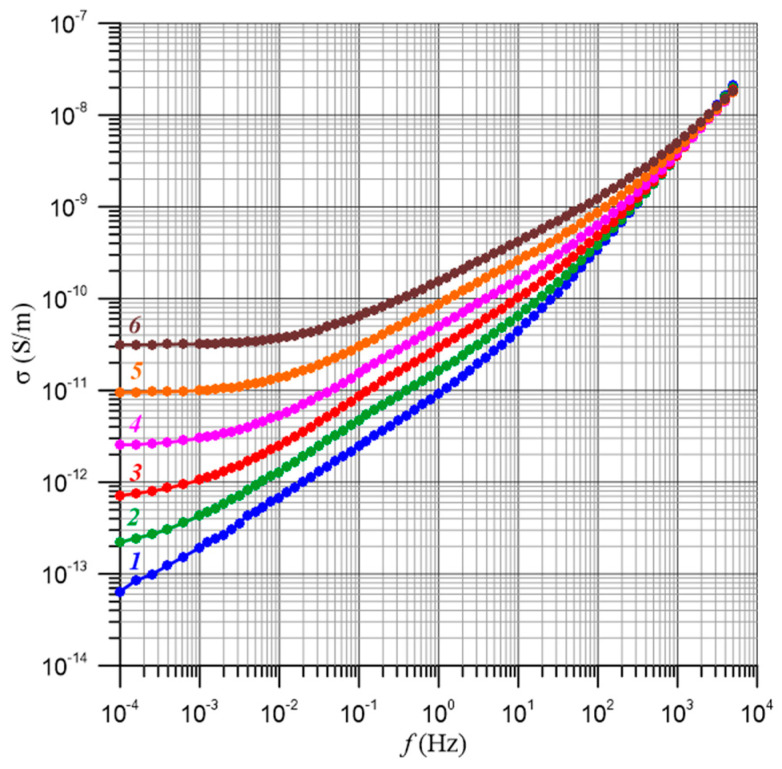
Frequency dependence of the conductivity of a pressboard with a moisture content of 2% by weight impregnated with bio-oil for temperatures: 1—20 °C; 2—30 °C; 3—40 °C; 4—50 °C; 5—60 °C; 6—70 °C.

Measurements of electrical parameters such as loss angle tangent, resistance, loss and capacitance at 50 Hz or 60 Hz, depending on the standard, have been used for many years in power transformer insulation diagnostics. The values of these parameters can be influenced not only by temperature, Formulas (24) and (25), but also by frequency. Therefore, when determining the activation energy at a specific frequency (50 Hz or 60 Hz), it is important to ensure that we are only dealing with the effect of temperature. If it is found that there is also a frequency dependence of the activation energy, this would mean that in Formula (24), the numerical factor σ_0_ is a function of frequency. This means that, in this case, the determined value of the activation energy cannot be used to convert the results of the measurements of the electrical insulation parameters to the reference temperature. Dependence relationships for a composite with a moisture content of 2% by weight are shown in Figure 4.

An Arrhenius plot was developed to directly determine the activation energy for each of the 73 measurement frequencies *f*_i_.

**Figure 5:**
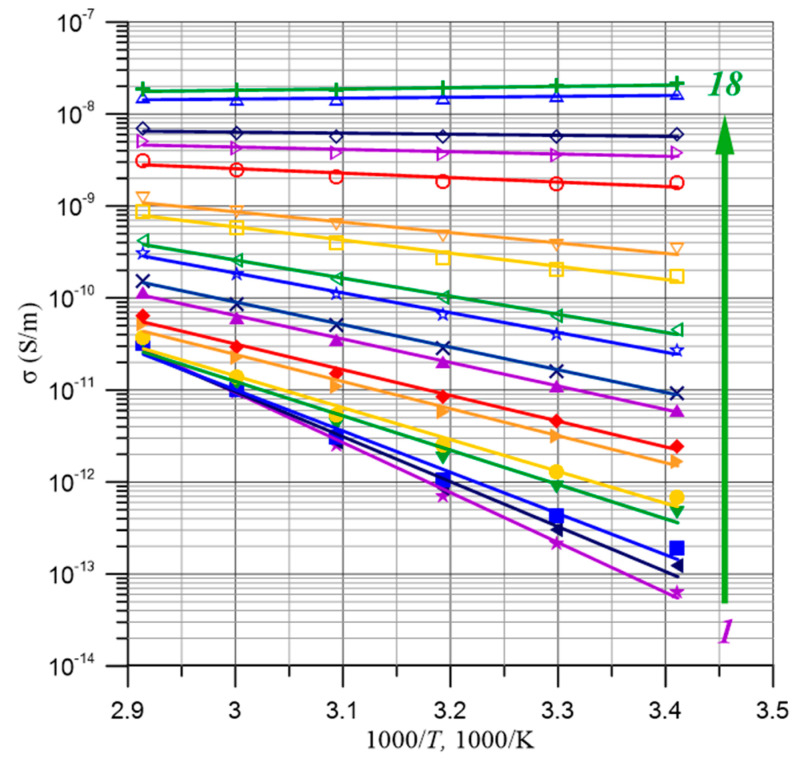
Arrhenius plots for the conductivity of pressboard with a moisture content of 2% by weight for 18 selected measurement frequencies *f*_i_ from 1—frequency 10^−4^ Hz—to 18—frequency 5·10^3^ Hz.

**Figure 6:**
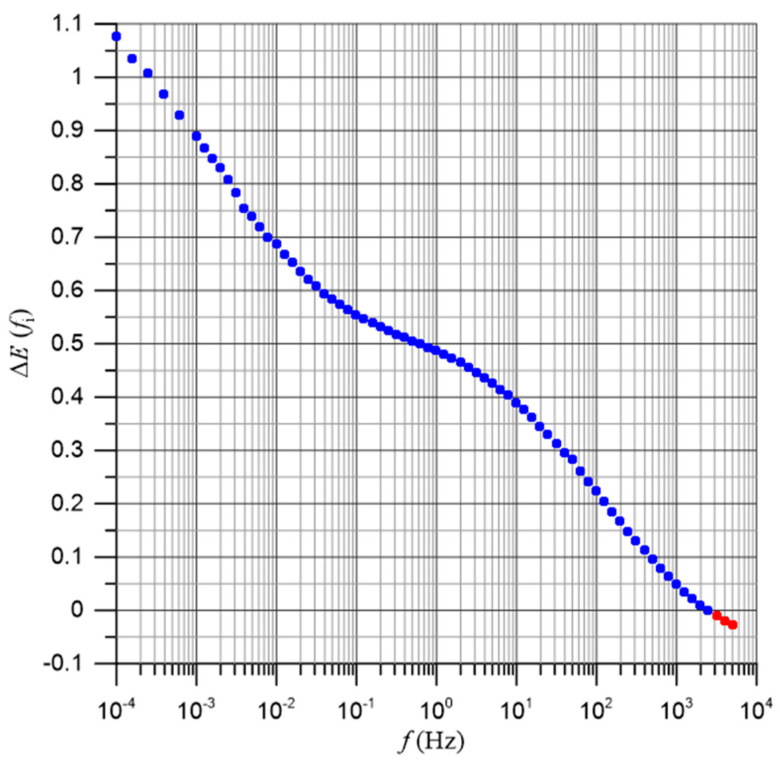
Frequency dependence of the activation energy of the conductivity of the bio-oil-impregnated pressboard with a moisture content of 2% by weight. Negative values are shown in red.

Based on linear approximations of the measurement results, activation energy values were determined for 73 measurement frequencies *f*_i_. Figure 5 shows 18 selected Arrhenius plots for measurement frequencies from 10^−4^ Hz to 5·10^3^ Hz. Figure 6 shows the frequency dependence of the activation energy for all 73 measurement frequencies *f*_i_.

It can be seen from Figure 6 that there is a frequency dependence of the activation energy in the composite, determined directly from the conductivity measurements for each of the applied measurement frequencies *f*_i_. The value of the activation energy thus determined decreases with increasing frequency. For the highest frequency values, its values become negative. In Figure 6, negative values are marked in red. Negative activation energy values are characteristic of conductivity in metals. It is difficult to imagine that a good insulating material such as pressboard impregnated with bio-oil would exhibit the characteristics typical of metallic conductivity. Therefore, it must be considered that the method used above to determine the activation energy of conductivity by determining it for specific measurement frequencies leads to erroneous conclusions, inconsistent with the insulating properties of the composite.

We will further analyze the conductivity and relaxation times of the composite based on the frequency–temperature relationships obtained for six samples with water contents ranging from 0.6% by weight to 5% by weight.

**Figure 7:**
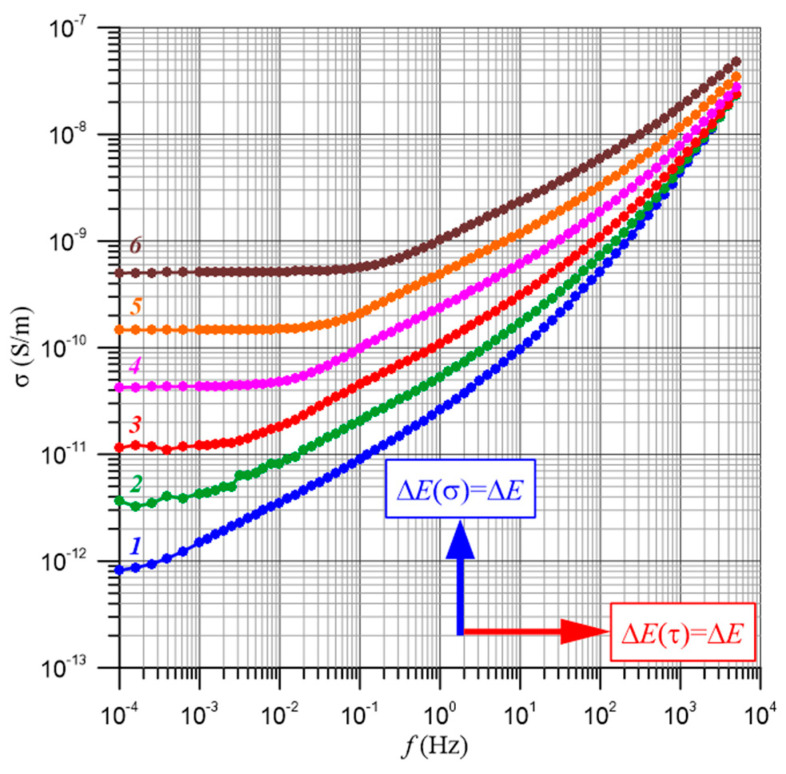
Frequency dependence of the conductivity of the composite pressboard—bio-oil—moisture for a water content of 4% by weight. Measurement temperatures: 1—20 °C; 2—30 °C; 3—40 °C; 4—50 °C; 5—60 °C; 6—70 °C.

**Figure 8:**
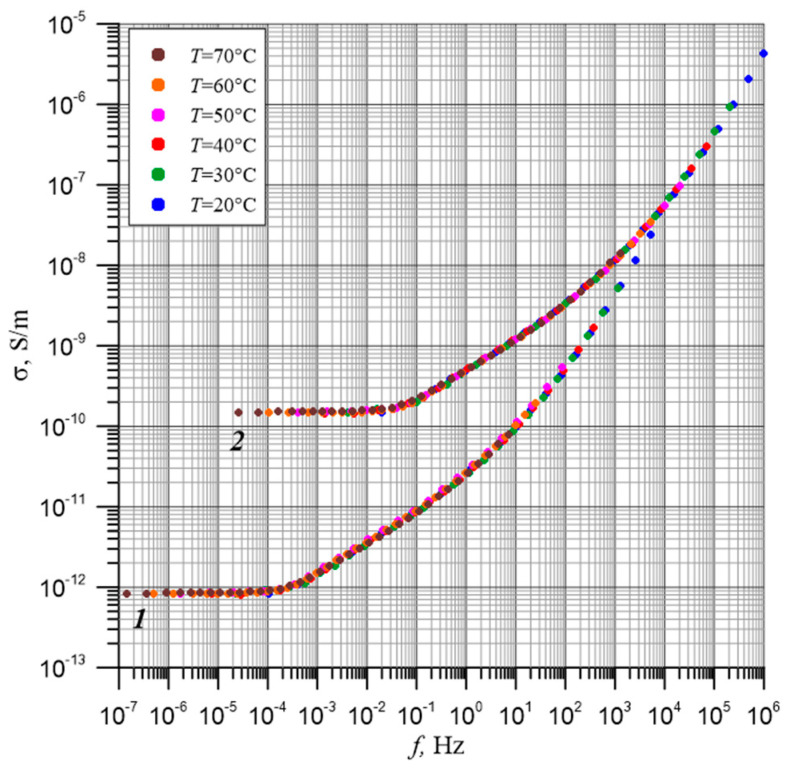
Shift in the σ(*f*, *T*) dependence along the X and Y axes using the generalized activation energy for moisture content of 4% by weight: 1—reference temperature 20 °C [61]; 2—reference temperature 60 °C. Every third point is marked on the graphs for each temperature.

Figure 7 shows, as an example, the frequency–temperature dependence of the conductivity of a composite of pressboard–bio-oil–water nanodroplets with a moisture content of 4% by weight. In [61], it was found that the relationships shown in Figure 7 and similar ones for other moisture contents are simultaneously influenced by two factors. The first is the temperature dependence of the conductivity. This relationship is referred to as the activation energy of conductivity Δ*E*(*σ*). Changes in temperature, according to Equation (25), shift the waveform along the Y axis. The second factor is related through the activation energy of the relaxation time Δ*E*(*τ*) to the temperature dependence of the relaxation time. The effect of temperature amounts to a shift in the waveform along the X axis. The shift is described by Equation (25). In [61], it was established that in pressboard–bio-oil–water nanodroplet composites, hopping conductivity is observed by electron tunneling between water nanodroplets. Such a conductivity mechanism causes the values of the activation energy of conductivity and relaxation times to be the same within the uncertainty limits of their determination and the value of the generalized activation energy Δ*E* to be as follows [61]:

(26) ΔE≈ΔE(σ)≈ΔE(τ)≈(1.02627±0.01606) eV,

Using the Δ*E* value, it is possible to convert the frequency dependence of conductivity obtained at any temperature to the selected reference temperature. For this purpose, the reference temperature curve is left in its place. The curves for higher temperatures are shifted down and to the left using the value of the Δ*E*. The curves for lower temperatures are shifted in a similar way up and to the right. The results of the conversion of the curves obtained for the sample with a water content of 4% by weight for the reference temperatures of 20 °C and 60 °C are shown in Figure 8. As can be seen from Figure 8, for both reference temperatures, the shifted curves overlap perfectly. From Figure 8, it can also be seen that the positions of the shifted curves in relation to the coordinates depend on the reference temperature chosen by us.

The shape of the curve depends only on the moisture content, concentrated in the composite in the form of nanodroplets. Similar results were obtained in [61] after recalculating the curves for moisture content from 0.6% by weight to 5% by weight to 20 °C. Based on the excellent overlap of the conductivity curves recalculated to any reference temperature established in [61] and in this work, it can be stated that:

- Firstly, the values of the activation energy of conductivity and the relaxation time are the same;
- Secondly, the value of the generalized activation energy does not depend on the measurement frequency in the range from 10^−4^ Hz to 5·10^3^ Hz;
- Thirdly, its value does not depend on the temperature in the tested range from 20 °C to 70 °C;
- Fourthly, and probably most importantly, the value of the generalized activation energy does not depend on the moisture content in the range from 0.6% by weight to 5% by weight.

As is known, the value of the relaxation time cannot be directly determined based on the study of electrical properties. Another important conclusion can be drawn from the finding that the values of the activation energy of conductivity and the relaxation time are identical. Namely, in the case of tunneling, the same value of the activation energy enters the formulas for the relaxation time and conductivity. In [24], it was established that the relaxation time in the case of electron tunneling is described by the following formula:

(27) $$\tau =\tau_{0}\exp\left(\frac{2}{R_{B}}r\right)\exp\frac{\Delta E}{kT},$$

The conductivity formula is as follows [36]:

(28) $$\sigma =\sigma_{0}\exp\left(-\frac{2}{R_{B}}r\right)\exp\left(-\frac{\Delta E}{kT}\right),$$

A comparison of Formulas (27) and (28) shows that the first exponents have the same values $\frac{2}{R_{B}}r$ and the second $\frac{\Delta E}{kT}$. Using the constancy of the activation energy of the conductivity value, we can eliminate its influence on the conductivity curves for different moisture contents. For this purpose, the same measurement temperature should be selected for the pressboard–bio-oil–water nanodroplets composite samples for different water contents X % by weight:

(29) $$\sigma \left(X,f,T=const\right)=\sigma_{0}\exp\left(-\frac{2}{R_{B}}r(X)\right)\exp\left(-\frac{\Delta E}{kT}\right)=\sigma_{0A}\left(T=const\right)\exp\left(-\frac{2}{R_{B}}r(X)\right),$$

where $\sigma_{0A}\left(T=const\right)=\sigma_{0}\exp\left(-\frac{\Delta E}{kT}\right)$.

**Figure 9:**
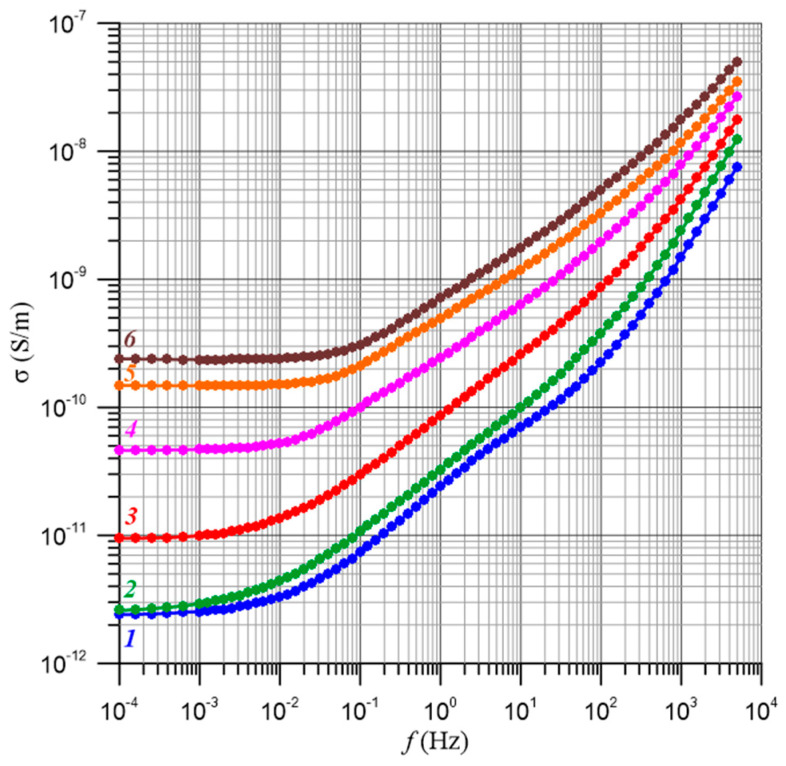
Frequency dependences of conductivity for water contents: 1—0.6% by weight; 2—1% by weight; 3—2% by weight; 4—3% by weight; 5—4% by weight; 6—5% by weight. Temperature of 60 °C.

Figure 9 shows the frequency dependence of conductivity for moisture contents from 0.6% by weight to 5% by weight measured at 60 °C. The temperature value of 60 °C was chosen because the curves have the lowest uncertainties in conductivity measurements.

The selection of a constant temperature for all moisture contents allowed for the elimination of the temperature’s influence, leaving the dependency on the moisture content through the average tunneling distance *r*(*X*) and the Bohr radius *R*_B_, which are included in Equation (29).

From Figure 2, it can be seen that the generalized activation energy Δ*E* corresponds to the transition of an electron from the state with *j =* 1 to the state with *k =* 2. From quantum mechanics, it follows that the relationship between the Bohr radius and the activation energy of conductivity is described, in this case, by the following equation [24]:

(30) $$R_{B}=\frac{h}{\pi }\sqrt{\frac{3}{32m_{e}\Delta E}},$$

From Equation (30), it follows that, in the case of the observed independence of activation energy from moisture content, the value of the Bohr radius also does not depend on the moisture content in the composite. This means that in Equation (29), which describes the dependence of conductivity on moisture content, the only remaining variable is *r*(*X*)—the average distance over which the electron tunnels. This distance is a function of the concentration of nanodroplets and, consequently, the moisture content.

For the constant temperature of 60 °C shown in Figure 9 and the constant activation energy, Equation (27) can be rewritten as follows:

(31) $$\tau \left(f,T=const\right)=\tau_{0A}\left(T=const\right)\exp\left[\frac{2}{R_{B}}r_{n}\left(X\right)\right],$$

where $\tau_{0A}\left(T=const\right)=\tau_{0}\exp\left(\frac{\Delta E}{kT}\right)$; *r_n_*(*X*)—the distance over which electrons tunnel between nanodroplets, each containing *n* molecules of water.

The distances over which electrons tunnel in the case of potential wells formed by individual water molecules are calculated according to the following formula [30]:

(32) $$r\left(X\right)=N^{-\frac{1}{3}}={\left(\frac{X\rho }{100uM_{H2O}}\right)}^{-\frac{1}{3}},$$

where *N*—concentration of water molecules per unit volume; *ρ*—mass density of water; *M_H_*_2*O*_ = 18—molar mass of water; *u* = 1.67 × 10^−27^ kg—atomic mass unit; *X*—moisture content, % by weight.

Molecules of water accumulate in the pressboard–bio insulating oil–moisture composite in the form of nanodroplets [29]. An increase in the number of water molecules in the nanodroplets results in longer distances between them. When there is an average of *n*_w_ water molecules in the nanodroplets, Formula (31) for the distance between their centers takes the following form:

(33) $$r_{n}\left(X\right)={\left(\frac{X\rho }{100unM_{H2O}}\right)}^{-\frac{1}{3}}=\sqrt[{3}]{n}r\left(X\right),$$

where *n*—the average number of water molecules in the nanodroplets.

In Formula (29), it should be taken into account that in the case of nanodroplets, the distance over which electrons tunnel increases from *r*(*X*)—Formula (32)—to *r_n_*(*X*)—Formula (33). The average number of water molecules in the nanodroplet *n* for the studied pressboard–bio-oil nanocomposite is not precisely defined. Therefore, Formula (29) should be transformed into the following expression:

(34) $$\sigma \left(X,f,T=const\right)=\sigma_{0A}\left(T=const\right)\exp\left(-B\left(f\right)r(X)\right),$$

where *B*(*f*)—a coefficient dependent on frequency; *r*(*X*)—the distance between water molecules, as seen in Formula (31).

Based on Formula (34) and the experimental waveforms presented in Figure 9, the values of the coefficient *B*(*f*) can be determined. To carry this out, we will logarithmize Formula (34):

(35) $$\ln\left[\sigma \left(X,f,T=const\right)\right]=\ln\left[\sigma_{0A}\left(T=const\right)\right]-B\left(f\right)r(X),$$

By plotting the dependence of the logarithm of conductivity for a selected measurement frequency as a function of the distance, defined by Formula (31), the value of the coefficient *B*(*f*) can be determined from the slope of the waveform. In this study, graphs of the dependencies described by Formula (35) were created in a semi-logarithmic scale for all 73 measurement frequencies using the data presented in Figure 9.

**Figure 10:**
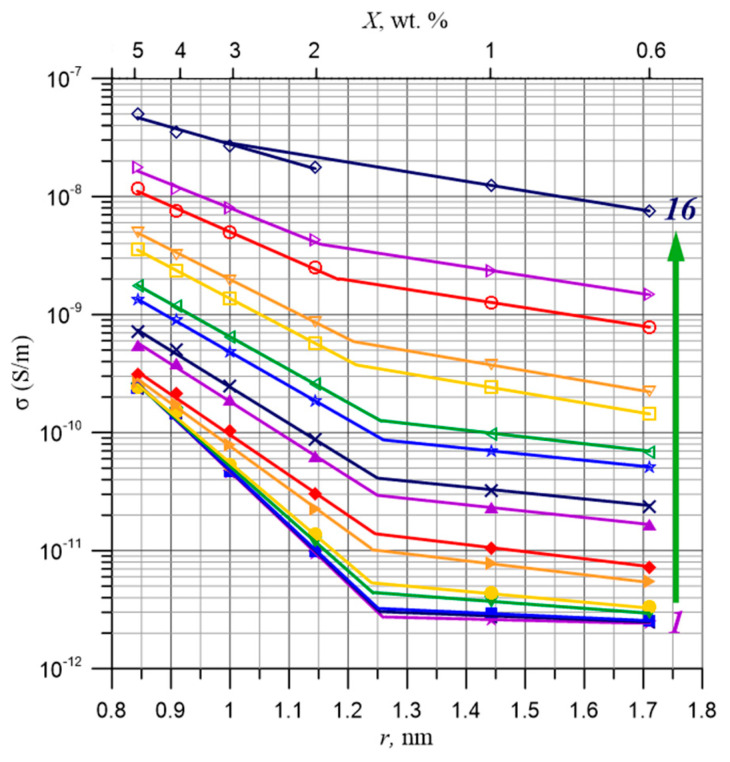
The dependencies of the conductivity of the pressboard–bio-oil–water nanodroplets composite on the distance between water molecules, according to Formula (32), for 16 selected frequencies ranging from (1)—10^−4^ Hz to (16)—5·10^3^ Hz. Measurement temperature: 60 °C.

**Figure 11:**
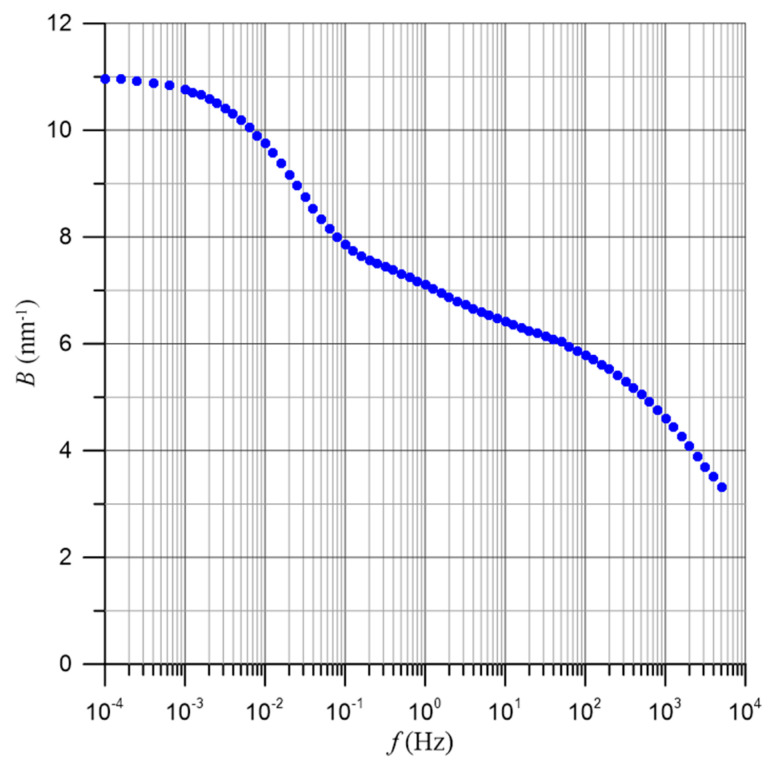
Frequency dependence of the coefficient value *B*(*f*), as shown in Formula (34).

Figure 10 shows the dependencies of the conductivity of the pressboard–bio-oil–water nanodroplets composite on the distance between water molecules for 16 selected frequencies ranging from 10^−4^ Hz to 5·10^3^ Hz. From Figure 10, it is evident that the curves of the dependency σ(*f*), especially for low frequencies, consist of two segments of straight lines. For low moisture contents, this segment is practically parallel to the X-axis. For higher water contents, there is a sharp increase in conductivity. Such characteristic waveforms are typical of the conductivity dependencies of composites and nanocomposites (see, for example, [62,63]). Below the percolation threshold, the conductivity is a constant value close to the conductivity of the dielectric matrix. After surpassing the percolation threshold, conductivity begins to increase rapidly. From the intersection of the flat segment and the steep segment for the low-frequency range, the value of the percolation threshold was determined to be *x*_c_ ≈ (1.4 ± 0.3)% by weight. From the approximation of the results obtained for moisture contents above the percolation threshold (2% by weight and greater), the values of the coefficient *B*(*f*) were determined for all 73 measurement frequencies. These values are presented in Figure 11.

Comparing the formulas for conductivity (28) and relaxation time (29) at a constant temperature, we see that both formulas include the same coefficient *B*(*f*). Using Formula (34) and the values of *B*(*f*) presented in Figure 11, the frequency-dependent relationships of the relative values of AC relaxation times were determined using the following formula:

(36) $$\tau_{ref}\left(f,X,T=const\right)=\frac{\tau \left(f,X,T=const\right)}{\tau_{0}\exp\left(\frac{\Delta E}{kT}\right)}=\exp\left[B\left(f\right)r\left(X\right)\right],$$

**Figure 12:**
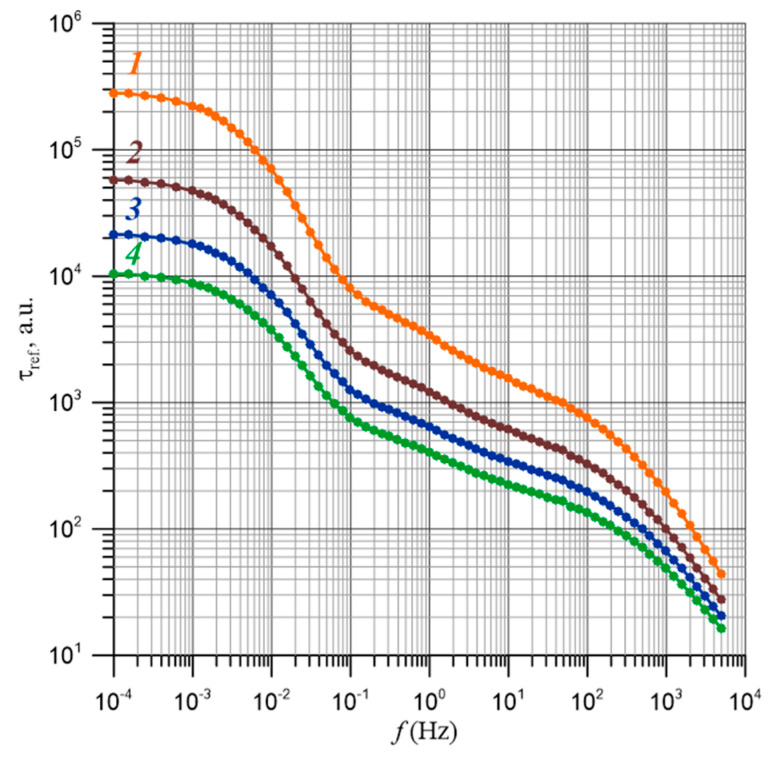
Frequency-dependent relationship of the relative relaxation time. 1—water content 2% by weight; 2—3% by weight; 3—4% by weight; 4—5% by weight.

The frequency-dependent relationships of the relative relaxation times for moisture content ranging from 2% by weight to 5% by weight are presented in Figure 12. The uncertainty in determining the relative relaxation time is approximately ±14%. For composites with a composition below the percolation threshold, there are no percolation channels. Therefore, the waveforms in Figure 12 show the relative relaxation times for water content above the percolation threshold, starting from 2% by weight. From Figure 12, it is evident that an increase in moisture content leads to a decrease in the relative relaxation times. According to Formula (27), as the temperature rises, the values of the relaxation times also decrease.

In the dependence τ_ref_ (*f*) (Figure 12), two distinct stages of relaxation time reduction are visible. One is located in the frequency range of approximately 10^−2^ Hz, while the next is around 10^3^ Hz. To accurately determine the position of the relaxation time reduction stages, numerical differentiation was performed according to the following formula:

(37) $$\frac{d\left[\log\tau_{ref}\left(f\right)\right]}{d(\log f)}\approx \frac{\log\tau_{ref}(f_{i+1})-\log\tau_{ref}(f_{i})}{\log f_{i+1}-\log f_{i}},$$

where *f_i_*—the measurement frequencies used in this study.

**Figure 13:**
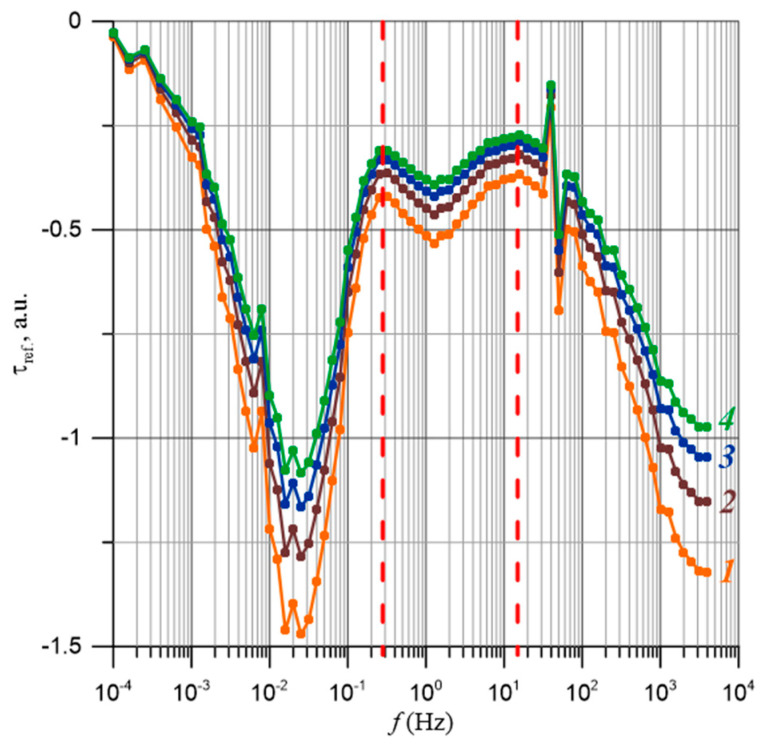
The frequency-dependent derivative of the logarithm of the relaxation time with respect to the logarithm of frequency: 1—*X* = 2% by weight; 2—3% by weight; 3—4% by weight; 4—5% by weight.

The results of the differentiation are presented in Figure 13. The waveforms shown in Figure 13 reveal three minima, indicating the positions of the stages of relaxation time reduction. Two of these minima are relatively pronounced and are also visible in the τ_ref_(*f*) dependence in Figure 12. However, the stage located between them is comparatively weak, making its observation in the curves from Figure 12 practically impossible. Only by applying numerical differentiation is it possible to observe this stage and determine its position. The areas corresponding to these stages can be delineated based on the positions of the maxima separating the minima, which are characteristic for each stage. The positions of the maxima are indicated in Figure 13 with vertical lines. The low-frequency stage of relaxation time reduction is found in the range from 10^−4^ Hz to approximately 3·10^−1^ Hz, while the second stage ranges from about 3·10^−1^ Hz to approximately 1.5·10^1^ Hz. As for the third stage, its starting point can only be determined at a frequency of about 1.5·10^1^ Hz. The end of this stage lies beyond the upper limit of the FDS meter, which is 5·10^3^ Hz. Assuming that the shape of the derivative for the third stage is somewhat symmetrical, similar to the other two stages, its end may be around a frequency in the order of 10^6^ Hz.

The constancy of the activation energy within the studied frequency range indicates that all three stages of relaxation time reduction are associated with the water nanodroplets. The presence of three stages of relaxation time reduction implies that the composite contains potential wells created by the nanodroplets of water, differing in their probability distribution of distances between nearest neighbors. The first stage corresponds to a normal distribution with a relatively high expected distance. For the second stage, the expected value is lower, while the smallest expected values occur for the third stage. It should be noted that the high-frequency dielectric permittivities determined for the medium in which the electrons tunnel, namely the bio-oil, are approximately 4 and about 1.9, respectively. This indicates that the nanodroplets are located not in the bio-oil but in the cellulose.

Based on the determined values of relative relaxation times, it is possible to define the ranges of changes in distances over which electrons tunnel at each stage. Namely, Equations (32) and (36) describe the same value of τ_ref_(*f,X,T = const*). This means that we can equate their right sides, which gives us:

(38) $$\exp\left[B\left(f\right)r\left(X\right)\right]=\exp\left[\frac{2}{R_{B}}r_{n}\left(X\right)\right],$$

From Equation (38), it follows that the distance over which electrons tunnel between the nanodroplets is related to the distance between individual water molecules, determined by the following expression:

(39) $$r_{n}\left(X\right)=r\left(X\right)B\left(f\right)\frac{R_{B}}{2}$$

where *r*(*X*)—the distance between individual water molecules; *B*(*f*)—the frequency-dependent coefficient shown in Figure 11; *R*_B_—the Bohr radius of the tunneling electron

**Figure 14:**
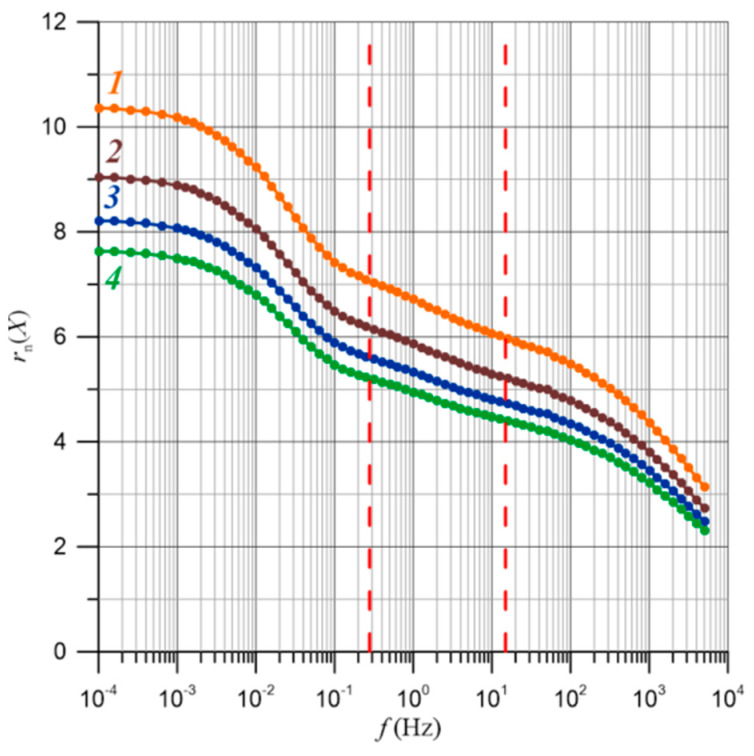
Frequency-dependent distances over which electrons tunnel. 1—water content 2% by weight; 2—3% by weight; 3—4% by weight; 4—5% by weight.

The value of the Bohr radius was determined from Equation (30) for electrons tunneling between water nanodroplets in pressboard impregnated with bio-oil. It is 1.65 nm. Using Equation (39), the values of *B*(*f*)—from Figure 11—and the value of *R_B_*, the frequency-dependent distances over which electrons tunnel between the nanodroplets, were calculated and are presented in Figure 14. In this figure, vertical lines indicate the boundaries between the three stages of relaxation time reduction, determined in Figure 13 based on the positions of the derivative maxima.

**Figure 15:**
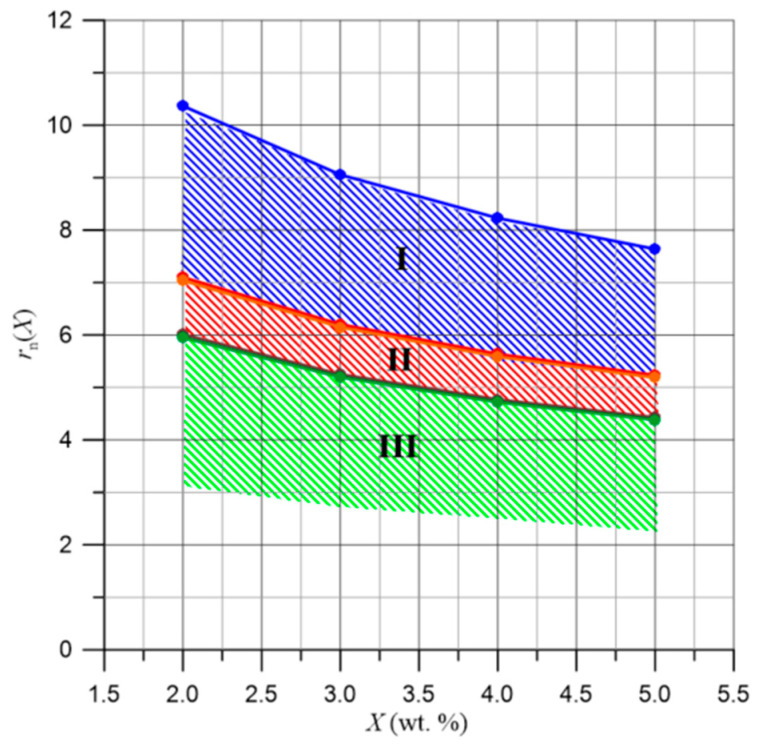
The dependencies of the distances over which electrons tunnel with respect to moisture content for stages I, II, and III are as follows: 1—the upper boundary of stage I is defined at a frequency of 10^−4^ Hz. 2—the upper boundary of stage II and the lower boundary of stage I are defined at a frequency of 3·10^−1^ Hz. 3—the upper boundary of stage III and the lower boundary of stage II are defined at a frequency of 1.5·10^1^ Hz.

Based on the results presented in Figure 14, the ranges of distances over which electrons tunnel for different water contents were determined and are shown in Figure 15.

In Figure 15, the boundaries of the distances over which electrons tunnel are shown for stages I and II with moisture content ranging from 2% by weight to 5% by weight. For stage I, the range of changes is approximately 5 nm, while for stage II, it is about 2.6 nm. As can be seen, this stage has the lowest range of distances for electron tunneling. For stage III, only the upper boundary of the range is shown. The determination of the lower boundary is not possible because it lies outside the measurement range—Figure 13. Although the range cannot be specified in this case, it is expected to be no less than approximately 1.5 nm. It is evident that, for each range, there are varied values of the distances over which electrons tunnel, and their widths also differ. This may indicate that the nanodroplets, between which tunneling occurs and causes the flow of alternating current in these ranges, are located within different components of cellulose fibers. The presence of three stages in the frequency-dependent relaxation time can be explained by considering the fibrous structure of cellulose. As is known, individual cellulose chains form ordered structures with a diameter in the order of 5 nm, called micelles. Groups of micelles create microfibrils with diameters ranging from 10 to 30 nm, which, due to mutual interactions, form fibers with diameters ranging from 20 to 60 μm and lengths of 1 to 3 mm [64,65,66]. The nanodroplets located within micelles, microfibrils, and fibers can have varied distances over which electrons tunnel. These distances are defined by the dimensions of the individual components of cellulose.

**Figure 16:**
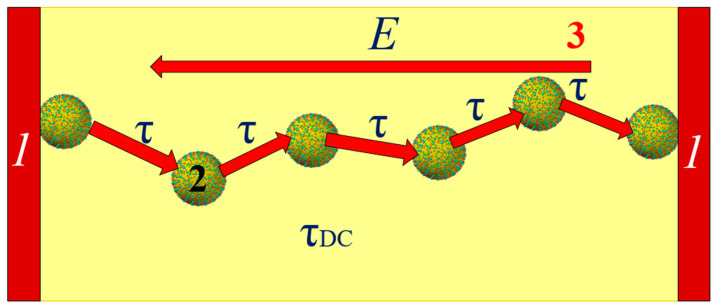
Schematic representation of the percolation channel for direct current flow: 1—electrodes; 2—nanodroplets; 3—vector of the electric field under direct voltage.

**Figure 17:**
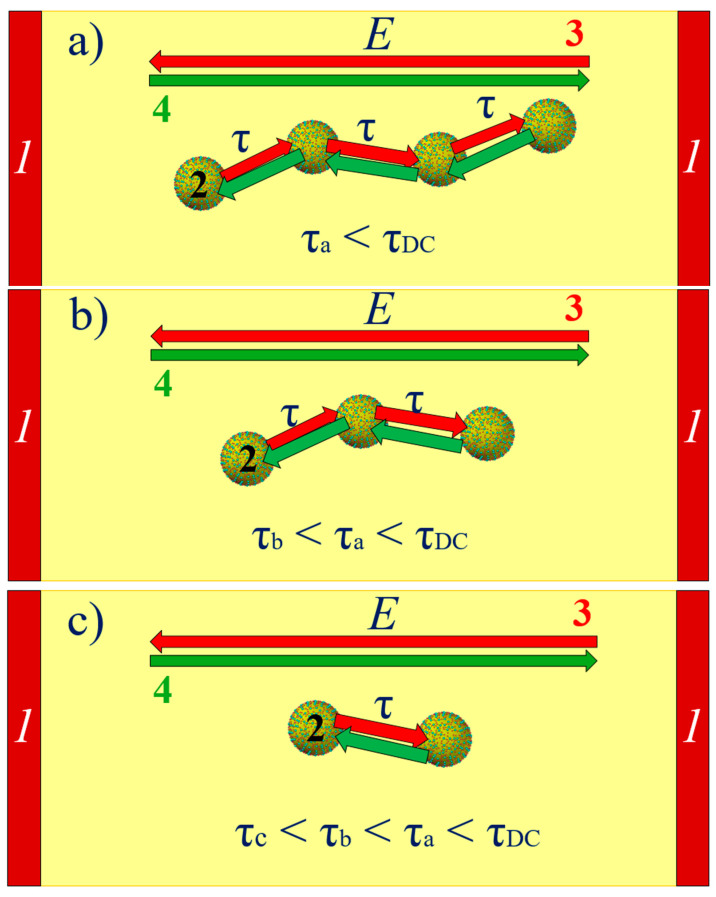
Schematic representation of alternating current flow in clusters with increased frequency—(**a**–**c**). 1—electrodes; 2—nanodroplets; 3—electric field vector in the first half-cycle; 4—electric field vector in the second half-cycle.

The frequency-dependent relationships of the relative relaxation times presented in Figure 12 can be explained as illustrated in Figure 16 and Figure 17.

At a constant voltage or at very low frequencies, the flow of current occurs through the percolation channel, connecting the electrodes. This is schematically presented in Figure 16. In the percolation channel, electron tunneling between neighboring nanodroplets occurs in the direction opposite to the electric field vector. Due to the length of the percolation channel and the presence of distances greater than the average, the relaxation time for direct current is the highest value.

In the transition to AC, a percolation channel is not required for conductivity. The transition to alternating current shortens the tunneling path because when the polarity of the electric field changes in the next half-cycle, the electron begins to tunnel in the opposite direction. This is illustrated in Figure 17a. Tunneling between the nanodroplets in one half-cycle is indicated by red arrows. After the voltage polarity changes in the next half-cycle, tunneling is shown with green arrows. Therefore, for conductivity, clusters are sufficient, the length of which is smaller than that of the percolation channel. These can be either segments of the percolation channel with distances between nanodroplets that are smaller than the average value or clusters consisting of multiple nanodroplets (see Figure 17a).

With further increases in frequency, the paths through which the electrons tunnel shorten (see Figure 17b). Ultimately, at high frequencies, tunneling occurs between pairs of neighboring nanodroplets (see Figure 17c).

## 5. Conclusions

In this study, the frequency–temperature dependence of the AC conductivity and relaxation times in electrotechnical pressboard intended for insulating power transformers was determined. The pressboard was impregnated with the innovative NYTRO^®^ BIO 300X bio-oil produced from plant raw materials. Tests were performed for six different water contents in the pressboard—from 0.6% by weight to 5% by weight in the frequency range from 10^−4^ Hz to 5·10^3^ Hz. Measurement temperatures ranged from 20 °C to 70 °C with a step of 10 °C.

The electrical conductivity of percolation channels in nanocomposites consisting of nanoparticles of a conductive phase in a dielectric matrix was analyzed. A composite of cellulose–bio insulating oil–water nanodroplets was chosen as an example. In such nanocomposites, DC conduction takes place via electron tunneling between the potential wells created by the water nanodroplets. A percolation channel connecting contacts to a material a few millimeters thick is formed by nanodroplets with dimensions in the order of a few nanometers. The number of nanodroplets in the channel can be up to 10^6^ or more. Based on the tunneling mechanism, the resistance of the percolation channel, in which the nanodroplets are randomly distributed, was calculated. According to the Central Limit Theorem, for such a large number of randomly distributed elements in the percolation channel, the normal probability distribution of the distance between neighboring nanodroplets was used for the calculation. The standard deviation from the mean value of the distance between adjacent nanodroplets was chosen as the measure of disorder. It was found that the percolation channel resistance value was lowest in the case of a regular distribution of nanodroplets. As disorder increases, the percolation channel resistance increases.

It was found that the experimental values of the activation energy of the conductivity and the relaxation time are the same within the limits of uncertainty and do not depend on the moisture content. This is another significant confirmation of the occurrence of current conduction via quantum mechanical electron tunneling phenomena in pressboard–bio-oil–water nanodroplet nanocomposites.

It was found that, in the lowest frequency region, the composite of pressboard–bio-oil–water conducts as with DC voltage. The conductivity value does not depend on the measurement frequency. As the frequency increases, the relaxation time decreases; so, the effect of moisture on the conductivity value decreases.

The dependence of the DC conductivity on the distance over which the electrons tunnel (moisture content) was determined. For low moisture contents, DC conductivity is practically constant. With a further increase in water content, there is a sharp increase in DC conductivity. Such curves are characteristic of the dependence of the DC conductivity of composites and nanocomposites on the conductive phase content. A percolation threshold value of *x*_c_ ≈ (1.4 ± 0.3)% by weight was determined from the intersection of horizontal and steeply sloping sections.

The frequency dependence of the values of the relative relaxation times was determined for composites with a moisture content from 2% by weight to 5% by weight, i.e., above the percolation threshold. The highest relative values of relaxation time τ_ref_ occur for DC and for the lowest frequencies close to 10^−4^ Hz. As the frequency increases, the relaxation time decreases. The derivatives d(*log*τ_ref_)/d(log*f*) were calculated, from the analysis of which it was determined that three stages of relaxation time decrease occur in the nanocomposites studied. The first occurs in the frequency region from 10^−4^ Hz to about 3·10^−1^ Hz, and the second from about 3·10^−1^ Hz to about 1.5·10^1^ Hz. The beginning of the third stage is at a frequency of about 1.5·10^1^ Hz. The end of this stage is above the upper range of the FDS meter of 5·10^3^ Hz, probably at frequencies of about 10^6^ Hz.

It was found that the nanodroplets are located in the cellulose and not in the bio-oil. The occurrence of three stages on the frequency dependence of the relaxation time can be explained when the fibrous structure of cellulose is taken into account. Nanodroplets, found in micelles, microfibrils and in the fibers of which cellulose is composed, can have varying distances between them, determined by the dimensions of these cellulose components.

## Data Availability

The original contributions presented in the study are included in the article, further inquiries can be directed to the corresponding author.

## References

[1] Krause C. (2012). Power transformer insulation—History, technology and design. IEEE Trans. Dielectr. Electr. Insul..

[2] Prevost T.A., Oommen T.V. (2006). Cellulose insulation in oil-filled power transformers: Part I—History and development. IEEE Electr. Insul. Mag..

[3] Martínez M., Pleite J. (2020). Improvement of RVM test interpretation using a Debye equivalent circuit. Energies.

[4] Fofana I., Wasserberg V., Borsi H., Gockenbach E. (2001). Retrofilling conditions of high-voltage transformers. IEEE Electr. Insul. Mag..

[5] Kurzweil P., Schell C., Haller R., Trnka P., Hornak J. (2021). Environmental Impact and Aging Properties of Natural and Synthetic Transformer Oils under Electrical Stress Conditions. Adv. Sustain. Syst..

[6] Lukenda N. (2019). Not all mineral oils are equal—Exploring the history and technology behind mineral insulating oils. Transform. Mag..

[7] Gielniak J., Graczkowski A., Moranda H., Przybylek P., Walczak K., Nadolny Z., Moscicka-Grzesiak H., Feser K., Gubanski S. (2013). Moisture in cellulose insulation of power transformers—Statistics. IEEE Trans. Dielectr. Electr. Insul..

[8] Oommen T.V., Prevost T.A. (2006). Cellulose insulation in oil-filled power transformers: Part II—Maintaining insulation integrity and life. IEEE Electr. Insul. Mag..

[9] Raetzke S., Koch M., Krueger M., Talib A. (2013). Condition assessment of instrument transformers using Dielectric Response Analysis. Elektrotechnik Und Informationstechnik.

[10] Fabre J., Pichon A. (1960). Deteriorating Processes and Products of Paper in Oil Application to Transformers. Proceedings of the International Conference on Large High Voltage Electric Systems.

[11] Nejedly J., Newesely G. (2004). Evaluation of the Extent of Ageing of Paper in Oil-Immersed. CIGRE Symp. Paris, Proceedings Paper D1-302.

[12] Lelekakis N., Martin D., Guo W., Wijaya J., Lee M. (2012). A field study of two online dry-out methods for power transformers. IEEE Electr. Insul. Mag..

[13] Martin D., Perkasa C., Lelekakis N. (2013). Measuring paper water content of transformers: A new approach using cellulose isotherms in nonequilibrium conditions. IEEE Trans. Power Deliv..

[14] Mishra D., Haque N., Baral A., Chakravorti S. (2017). Assessment of interfacial charge accumulation in oil-paper interface in transformer insulation from polarization-depolarization current measurements. IEEE Trans. Dielectr. Electr. Insul..

[15] Hao J., Liao R., Chen G., Ma Z., Yang L. (2012). Quantitative analysis ageing status of natural ester-paper insulation and mineral oil-paper insulation by polarization/depolarization current. IEEE Trans. Dielectr. Electr. Insul..

[16] Zhang S.L. The simulation analysis of transformer recovery voltage by field and circuit method based on PSO algorithm. Proceedings of the 2018 12th International Conference on the Properties and Applications of Dielectric Materials (ICPADM).

[17] Zhang T., Li X., Lv H., Tan X. (2014). Parameter Identification and Calculation of Return Voltage Curve Based on FDS Data. IEEE Trans. Appl. Supercond..

[18] Fofana I., Yéo Z., Farzaneh M. (2009). Dielectric response methods for diagnostics of power transformers. Recent Adv. Dielectr. Mater..

[19] Brncal P., Gutten M. (2021). Diagnostics of Insulating Condition of Traction Transformer by Frequency Method. Transp. Res. Procedia.

[20] Zhao H., Mu H., Zhang D., DIng N., Wu Y., Zhang G., Tian J., Liang Z. (2021). Analysis of FDS Characteristics of Oil-impregnated Paper Insulation under High Electric Field Strength Based on the Motion of Charge Carriers. IEEE Trans. Dielectr. Electr. Insul..

[21] Wen H., Cheng L., Jiang Y., Zhu T., Chen Z., Dai X., Gao C. (2021). Influence of Water Molecules on Polarization Behavior and Time–Frequency Dielectric Properties of Cellulose Insulation. J. Electr. Eng. Technol..

[22] Fan X., Liu J., Lai B., Zhang Y., Zhang C. (2021). Fds measurement-based moisture estimation model for transformer oil-paper insulation including the aging effect. IEEE Trans. Instrum. Meas..

[23] Koch M., Tenbohlen S., Krüger M., Kraetge A. A Comparative Test and Consequent Improvements on Dielectric Response Methods. Proceedings of the XVth International Symposium on High Voltage Engineering, ISH.

[24] Żukowski P., Kierczyński K., Kołtunowicz T.N.N., Rogalski P., Subocz J. (2019). Application of elements of quantum mechanics in analysing AC conductivity and determining the dimensions of water nanodrops in the composite of cellulose and mineral oil. Cellulose.

[25] Zukowski P., Rogalski P., Kołtunowicz T.N., Kierczynski K., Zenker M., Pogrebnjak A.D., Kucera M. (2022). DC and AC Tests of Moisture Electrical Pressboard Impregnated with Mineral Oil or Synthetic Ester-Determination of Water Status in Power Transformer Insulation. Energies.

[26] (2020). NYTRO®BIO 300X–The New Bio-Based Alternative from Nynas. Transform. Technol. Mag..

[27] Fritsche R., Geissler M., Pukel G., Wolmarans C. The use of a Bio-Based Hydrocarbon Insulating Liquid in Power Transformers. Proceedings of the 2022 IEEE 21st International Conference on Dielectric Liquids (ICDL).

[28] Rozga P., Stuchala F., Pahlanvapour B., Wolmarans C. Lightning Impulse Breakdown Characteristics of a Bio-Based Hydrocarbon and Other Insulating Liquids under Positive Polarity. Proceedings of the 2022 IEEE 21st International Conference on Dielectric Liquids (ICDL).

[29] Żukowski P., Kołtunowicz T.N., Kierczyński K., Subocz J., Szrot M. (2015). Formation of water nanodrops in cellulose impregnated with insulating oil. Cellulose.

[30] Zukowski P., Kołtunowicz T.N., Kierczyński K., Subocz J., Szrot M., Gutten M. (2014). Assessment of water content in an impregnated pressboard based on DC conductivity measurements theoretical assumptions. IEEE Trans. Dielectr. Electr. Insul..

[31] Shklovskii B.I., Efros A.L. (1984). Electronic Properties of Doped Semiconductors.

[32] Mamunya Y.P., Davydenko V.V., Pissis P., Lebedev E.V. (2002). Electrical and thermal conductivity of polymers filled with metal powders. Eur. Polym. J..

[33] Zukowski P., Kołtunowicz T., Partyka J., Fedotova Y.A., Larkin A.V. (2009). Electrical properties of nanostructures (CoFeZr)_x_ + (Al_2_O_3_)_1−x_ with use of alternating current. Vacuum.

[34] Kołtunowicz T.N., Zhukowski P., Fedotova V.V., Saad A.M., Larkin A.V., Fedotov A.K. (2011). The features of real part of admittance in the nanocomposites (Fe_45_Co_45_Zr_10_) × (Al_2_O_3_) 100× manufactured by the ion-beam sputtering technique with Ar ions. Acta Phys. Pol. A.

[35] Landau L.D., Lifshitz E.M. (1981). Quantum Mechanics: Non-Relativistic Theory.

[36] Mott N.F., Davis E.A. (1979). Electronic Processes in Non-Crystalline Materials.

[37] Zukowski P.W., Rodzik A., Shostak Y.A. (1997). Dielectric constant and ac conductivity of semi-insulating Cd1-xMnxTe semiconductors. Semiconductors.

[38] Żukowski P., Kołtunowicz T., Partyka J., Węgierek P., Komarov F.F., Mironov A.M., Butkievith N., Freik D. (2007). Dielectric properties and model of hopping conductivity of GaAs irradiated by H+ ions. Vacuum.

[39] Pogrebnjak A., Ivashchenko V., Maksakova O., Buranich V., Konarski P., Bondariev V., Zukowski P., Skrynskyy P., Sinelnichenko A., Shelest I. (2021). Comparative measurements and analysis of the mechanical and electrical properties of Ti-Zr-C nanocomposite: Role of stoichiometry. Measurement.

[40] Pollak M., Geballe T.H. (1961). Low-Frequency Conductivity Due to Hopping Processes in Silicon. Phys. Rev..

[41] Ravich Y.I., Nemov S.A. (2002). Hopping conduction via strongly localized impurity states of indium in PbTe and its solid solutions. Semiconductors.

[42] Gholipur R., Khorshidi Z., Bahari A. (2016). A Study on Double Negative Properties of Metal–Dielectric Nanocomposites. JOM.

[43] Jan R., Habib A., Abbasi H.Y. (2016). High aspect ratio graphene nanosheets cause a very low percolation threshold for polymer nanocomposites. Acta Phys. Pol. A.

[44] Münstedt H., Starý Z. (2016). Is electrical percolation in carbon-filled polymers reflected by rheological properties?. Polymer.

[45] Zeng R.T., Hu W., Wang M., Zhang S.D., Zeng J.B. (2016). Morphology, rheological and crystallization behavior in non-covalently functionalized carbon nanotube reinforced poly(butylene succinate) nanocomposites with low percolation threshold. Polym. Test..

[46] Nowak R. (2002). Statystyka dla Fizyków.

[47] Kudryashov M.A., Mashin A.I., Logunov A.A., Chidichimo G., De Filpo G. (2012). Frequency dependence of the electrical conductivity in Ag/PAN nanocomposites. Tech. Phys..

[48] https://weidmann-electrical.com/insulation-technology/transformerboard/cellulose-based/.

[49] Ekanayake C., Gubanski S.M., Graczkowski A., Walczak K. (2006). Frequency Response of Oil Impregnated Pressboard and Paper Samples for Estimating Moisture in Transformer Insulation. IEEE Trans. Power Deliv..

[50] Liao R., Liu J., Yang L., Gao J., Zhang Y., Lv Y.D., Zheng H. (2015). Understanding and analysis on frequency dielectric parameter for quantitative diagnosis of moisture content in paper-oil insulation system. IET Electr. Power Appl..

[51] Kouassi K., Fofana I., Cissé L., Hadjadj Y., Yapi K., Diby K. (2018). Impact of Low Molecular Weight Acids on Oil Impregnated Paper Insulation Degradation. Energies.

[52] Liu J., Fan X., Zhang Y., Zhang C., Wang Z. (2020). Aging evaluation and moisture prediction of oil-immersed cellulose insulation in field transformer using frequency domain spectroscopy and aging kinetics model. Cellulose.

[53] (1997). Insulating Liquids—Oil-Impregnated Paper and Pressboard—Determination of Water by Automatic Coulometric Karl Fischer Titration.

[54] Rogalski P. (2020). Measurement Stand, Method and Results of Composite Electrotechnical Pressboard-Mineral Oil Electrical Measurements. Devices Methods Meas..

[55] Zukowski P., Kierczynski K., Koltunowicz T.N., Rogalski P., Subocz J., Korenciak D. (2020). AC conductivity measurements of liquid-solid insulation of power transformers with high water content. Measurement.

[56] Zukowski P., Rogalski P., Koltunowicz T.N., Kierczynski K., Subocz J., Sebok M. (2021). Influence of temperature on phase shift angle and admittance of moistened composite of cellulose and insulating oil. Measurement.

[57] (2018). DIRANA—The Fastest Way of Moisture Determination of Power- and Instrument Transformers and Condition Assessment of Rotating Machines. *Omi. L2894*. https://pdf.directindustry.com/pdf/omicron-electronics/dirana-fastestway-moisture-determination-power-instrument-transformers-condition-assessment-rotating-machines/13971-42092.html.

[58] Landau L.D., Lifshitz E.M., Pitaevskii L.P. (1984). Electrodynamics of Continuous Media.

[59] Mott N.F., Gurney R.W. (1950). Electronic Processes in Ionic Crystals.

[60] Psarras G.C., Manolakaki E., Tsangaris G.M. (2003). Dielectric dispersion and ac conductivity in—Iron particles loaded—Polymer composites. Compos. Part A Appl. Sci. Manuf..

[61] Zukowski P., Kierczynski K., Rogalski P., Okal P., Zenker M., Pajak R., Szrot M., Molenda P., Koltunowicz T.N. (2024). Research on the Influence of Moisture in the Solid Insulation Impregnated with an Innovative Bio-Oil on AC Conductivity Used in the Power Transformers. Energies.

[62] Imry Y. (2002). Introduction to Mesoscopic Physics.

[63] Mott N.F. (1974). Metal-Insulator Transitions.

[64] Chinga-Carrasco G., Miettinen A., Luengo Hendriks C.L., Gamstedt E.K., Kataja M. (2011). Structural Characterisation of Kraft Pulp Fibres and Their Nanofibrillated Materials for Biodegradable Composite Applications. Nanocomposites Polym. Anal. Methods.

[65] Butchosa N., Leijon F., Bulone V., Zhou Q. (2019). Stronger cellulose microfibril network structure through the expression of cellulose-binding modules in plant primary cell walls. Cellulose.

[66] Etale A., Onyianta A.J., Turner S.R., Eichhorn S.J. (2023). Cellulose: A Review of Water Interactions, Applications in Composites, and Water Treatment. Chem. Rev..

